# Hijacking of host Src-PI3K-Akt signaling by WSSV IE1 protein suppresses apoptotic and autophagic defenses to facilitate viral proliferation

**DOI:** 10.1128/jvi.01676-25

**Published:** 2025-12-16

**Authors:** Kaiyu Lu, Jia Zhang, Jinghua Zhu, Yongzhen Zhao, Xiuli Chen, Yueling Zhang, Defu Yao

**Affiliations:** 1 Guangdong Provincial Key Laboratory of Marine Biology, Institute of Marine Sciences, Shantou University 12386, Shantou, China; 2 Guangxi Key Laboratory of Aquatic Genetic Breeding and Healthy Aquaculture, Guangxi Academy of Fishery Sciences 718457, Nanning, China; Wageningen University & Research, Wageningen, Netherlands

**Keywords:** WSSV, immediate-early protein, Src, PI3K-Akt pathway, immune evasion

## Abstract

The phosphoinositide 3-kinase (PI3K)-Akt pathway is a key signaling cascade regulating diverse cellular processes, including proliferation, survival, autophagy, translation, and metabolism. White spot syndrome virus (WSSV), a major pathogen devastating global crustacean aquaculture, has been demonstrated to exploit the PI3K-Akt pathway to facilitate its proliferation. However, the precise mechanism underlying this viral modulation remained unclear. In this study, we demonstrate that WSSV infection induces activation of the PI3K-Akt pathway during the early infection stage in *Penaeus vannamei*. Mechanistically, we reveal that the WSSV immediate-early protein IE1 interacts with and activates host Src64B kinase via its Y_129_FTS tyrosine motif. This specific interaction promotes recruitment of the PI3K regulatory subunit alpha (PI3Kp85α), thereby triggering the downstream PI3K-Akt signaling. By activating this pathway, WSSV establishes a favorable environment for its proliferation by suppressing host apoptotic and autophagic defenses. Our findings unveil a previously unknown mechanism of WSSV immune evasion through Src-PI3K-Akt signaling hijacking and identify components of this signaling hub as potential therapeutic targets for anti-WSSV strategies.

**IMPORTANCE:**

Viruses usually hijack host signaling pathways to enhance infectivity and evade immune defenses. Understanding these interactions is critical for elucidating viral pathogenesis and developing effective antiviral strategies. Here, we demonstrate that the WSSV immediate-early protein IE1 binds to and activates host Src64B kinase, which in turn recruits PI3Kp85α and activates the PI3K-Akt signaling cascade. Activation of this pathway suppresses apoptosis and autophagy, thereby facilitating viral proliferation. These findings advance our understanding of WSSV pathogenesis and identify the Src-PI3K-Akt signaling as a promising therapeutic target for anti-WSSV intervention.

## INTRODUCTION

White spot syndrome virus (WSSV) is the etiological agent of white spot disease (WSD), a highly lethal and contagious disease causing substantial economic losses in global crustacean aquaculture (1). WSSV exhibits broad host tropism, infecting numerous decapod species, including penaeid shrimp, crayfish, lobsters, and crabs, with penaeid shrimp being particularly susceptible (2). Classified by the International Committee on Taxonomy of Viruses (ICTV) as the sole species within the genus *Whispovirus* (family *Nimaviridae*) (3), WSSV is a large, enveloped virus with a double-stranded circular DNA genome, ranging from 280 to 307 kilobases (4–6). Despite extensive research over three decades (7, 8), key mechanisms of WSSV infection remain incompletely understood, and no effective therapeutics exist. Thus, investigating factors associated with WSSV infection is crucial for developing antiviral strategies.

Phosphoinositide 3-kinase (PI3K) is a heterodimeric lipid kinase comprising a regulatory subunit (p85) and a catalytic subunit (p110)(9). PI3K activation occurs when the SH2 domain of the p85 binds autophosphorylated receptor or nonreceptor tyrosine kinases at the plasma membrane. This binding activates p110, enabling phosphorylation of phosphatidylinositol-4,5-bisphosphate (PIP2) to generate phosphatidylinositol-3,4,5-trisphosphate (PIP3). PIP3 acts as a second messenger, recruiting pleckstrin homology (PH) domain-containing kinases like Akt. Akt subsequently undergoes dual phosphorylation—Thr308 (mediated by PDK1) and Ser473 (mediated by mTORC2)—to achieve full activation. Once activated, Akt translocates from the plasma membrane to various intracellular compartments, where it phosphorylates downstream substrates to regulate processes such as proliferation, survival, autophagy, translation, and metabolism (10, 11). Given its critical role in cellular homeostasis, the PI3K-Akt pathway is frequently hijacked by viruses to support infection. Emerging evidence highlights its multifaceted contributions to viral infections, such as facilitating viral entry, inhibiting apoptosis, enhancing viral protein synthesis, and boosting viral replication (12–14). In penaeid shrimp, studies have demonstrated that the PI3K-Akt pathway facilitates WSSV infection through several mechanisms. For instance, WSSV hijacks this pathway to induce aerobic glycolysis (the Warburg effect), thereby reprogramming host metabolism to fulfill its energy and biosynthetic demands (15). Additionally, this metabolic shift also helps maintain cellular redox balance, protecting against oxidative damage caused by reactive oxygen species during infection (16). Furthermore, WSSV leverages this pathway to induce lipid biosynthesis, which is crucial for viral morphogenesis (17). However, the exact molecular mechanism by which WSSV modulates the PI3K-Akt pathway in shrimp remains unclear.

The immediate-early (IE) genes of DNA viruses encode crucial regulatory proteins that facilitate viral infection. Among the 21 IE genes identified in WSSV (18–20), IE1, the most extensively studied, functions as a transcription factor, exhibiting transactivation and DNA-binding capabilities (21). IE1 also interacts with multiple host proteins, including Retinoblastoma protein (Rb)(22), signal transducer and activator of transcription (STAT) (23), c-Jun N-terminal kinase (JNK) (24), β-catenin (25), prophenoloxidase (proPO)(26), and integrin-α5(27) to modulate host signaling and enhance viral proliferation. Interestingly, our prior work indicated potential interactions between IE1 and components of the PI3K-Akt pathway, specifically Src64B and PI3Kp85α (26), suggesting IE1’s involvement in regulating this pathway. In this study, we clarify a mechanistic cascade in which IE1 binds and activates the Src64B via its Y_129_FTS tyrosine motif, promoting PI3Kp85α recruitment and PI3K-Akt pathway activation. This virus-induced Src-PI3K-Akt signaling suppresses host apoptotic and autophagic defenses, creating a favorable environment for WSSV proliferation. Our findings reveal a novel WSSV strategy for hijacking the host Src-PI3K-Akt signaling and identify promising targets for anti-WSSV intervention.

## RESULTS

### IE1 interacts with host Src64B and PI3Kp85α

**Fig 1:**
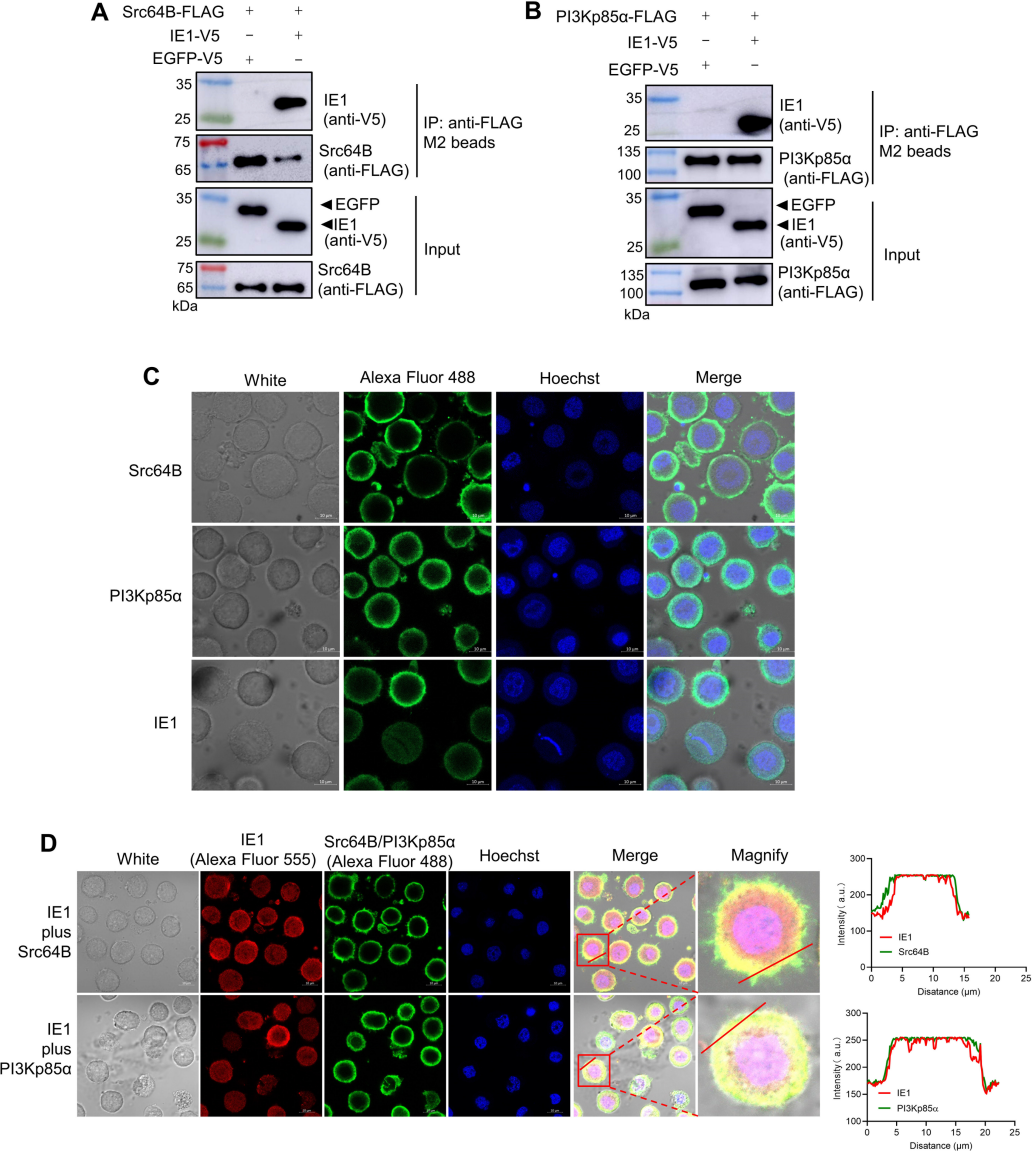
IE1 interacts with host Src64B and PI3Kp85α. (**A and B**) Co-IP analysis of IE1 binding to Src64B and PI3Kp85α. High Five cells were co-transfected with either Src64B-FLAG or PI3Kp85α-FLAG along with IE1-V5 (EGFP-V5 as control). At 48 h post-transfection, cell lysates were immunoprecipitated using anti-FLAG beads, and interactions were assessed using western blot. (**C**) Subcellular localization of IE1, Src64B, and PI3Kp85α. Cells transfected individually with IE1-V5, Src64B-FLAG, or PI3Kp85α-FLAG were fixed at 48 h and immunostained with anti-V5 or anti-FLAG antibodies. Scale bar, 10 µm. (**D**) IE1 co-localizes with Src64B and PI3Kp85α. Cells co-transfected with IE1-V5 and either Src64B-FLAG or PI3Kp85α-FLAG were subjected to dual immunofluorescence staining (anti-V5 and anti-FLAG) at 48 h. Co-localization was quantified using ImageJ. Scale bar, 10 μm.

Our previous proteomics data indicated potential interactions between WSSV IE1 and *Penaeus vannamei* Src64B and PI3Kp85α (26). Domain analysis revealed that *P. vannamei* Src64B is highly conserved, featuring characteristic Src family kinase domains: an Src homology 3 (SH3) domain, an SH2 domain, and a tyrosine kinase catalytic (TyrKc) domain (Fig. S1A). In contrast, *P. vanname*i PI3Kp85α shows lower conservation relative to its human ortholog. Notably, it contains two additional protein kinase C conserved region 1 (C1) domains and an N-terminal sterile alpha motif (SAM) domain, which are absent in *Homo sapiens* PI3Kp85 (Fig. S1B). To assess physical interactions, co-immunoprecipitation (Co-IP) assays in High Five cells demonstrated that ectopically expressed IE1 specifically binds both Src64B and PI3Kp85α (Fig. 1A and B). Immunofluorescence assays showed that individually expressed IE1 localized throughout the cytoplasm and nucleus, Src64B exclusively to the plasma membrane, and PI3Kp85α predominantly to the cytoplasm (Fig. 1C). However, upon co-expression, IE1 was observed to co-localize with Src64B and PI3Kp85α at the plasma membrane (Fig. 1D). These data demonstrate functional interactions between IE1 and both Src64B and PI3Kp85α.

### IE1 activates Src64B via a tyrosine motif to recruit PI3Kp85α

**Fig 2:**
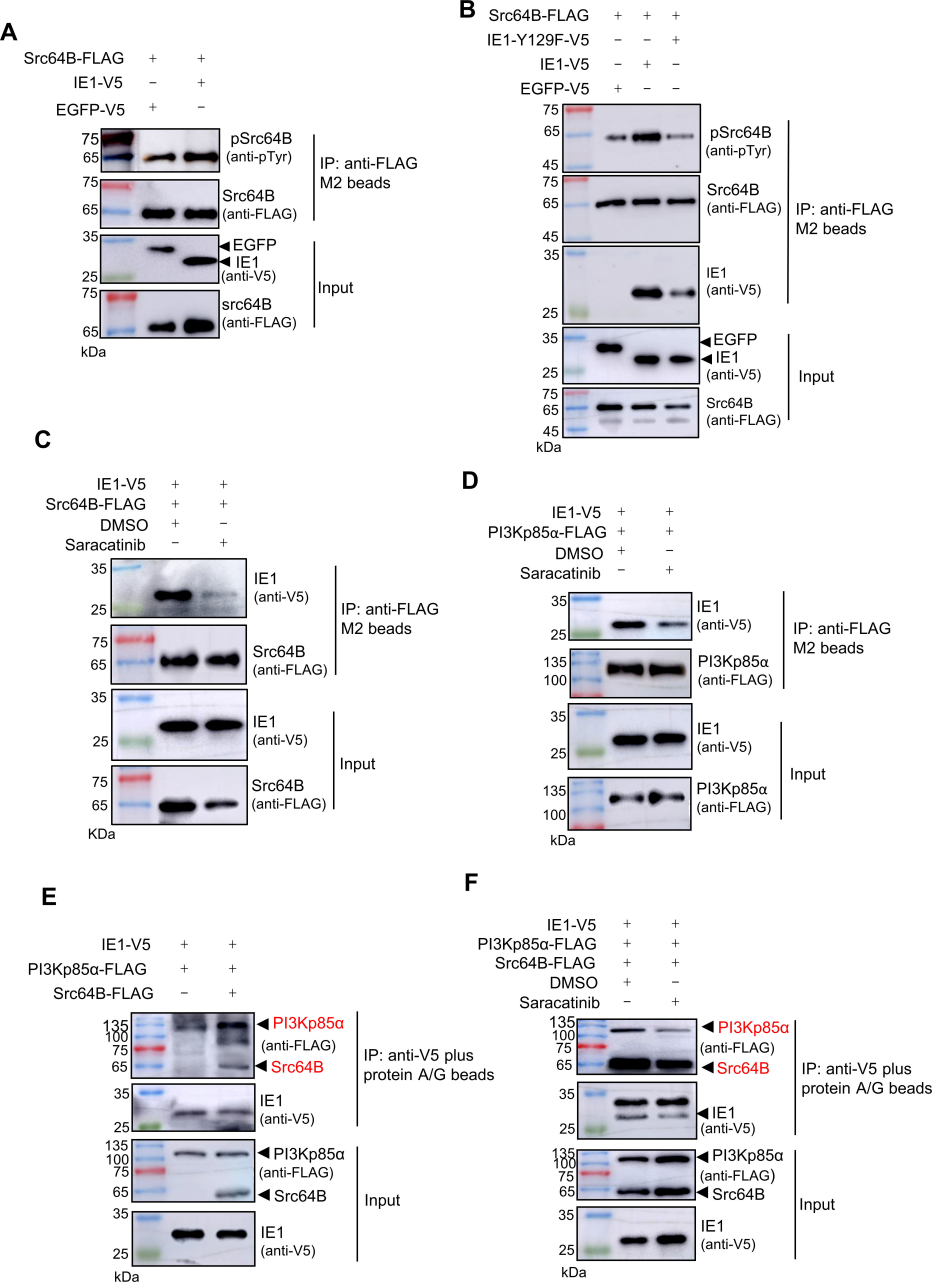
IE1 activates Src64B to recruit PI3Kp85α. (**A**) IE1 overexpression enhances Src64B activation. High Five cells were co-transfected with Src64B-FLAG and either IE1-V5 or EGFP-V5 (control). At 48 h post-transfection, Src64B-FLAG was immunoprecipitated (IP) using anti-FLAG beads, and tyrosine phosphorylation was assessed by western blot with an anti-phosphotyrosine antibody. (**B**) The Y_129_FTS tyrosine motif is critical for IE1-Src64B interaction and Src64B activation. Cells co-expressing Src64B-FLAG with either wild-type IE1-V5 or the mutant IE1-Y129F-V5 were subjected to co-IP using anti-FLAG beads at 48 h. IP samples were analyzed by western blot for IE1 binding and Src64B phosphorylation. (**C and D**) Saracatinib inhibits the binding of IE1 to Src64B and PI3Kp85α. Cells co-expressing IE1-V5 with either Src64B-FLAG (**C**) or PI3Kp85α-FLAG (**D**) were treated with 10 µM Saracatinib or DMSO (control) at 24 h. The interactions between IE1 and Src64B or PI3Kp85α were analyzed by co-IP at 48 h post-transfection. (**E**) Src64B overexpression enhances IE1-PI3Kp85α interaction. PI3Kp85α-FLAG and IE1-V5 were cotransfected with or without Src64B-FLAG, followed by Co-IP analysis at 48 h. (**F**) Saracatinib inhibits the formation of the IE1-Src64B-PI3Kp85α ternary complex. Cells triply transfected with IE1-V5, Src64B-FLAG, and PI3Kp85α-FLAG were treated with Saracatinib or DMSO (24 h) before co-IP analysis.

Viral proteins are able to bind and activate host Src family kinases via tyrosine motifs (28, 29). In this study, we identified a putative tyrosine motif at residue 129 of IE1 (Y_129_FTS), suggesting its potential to activate Src64B via binding. To test this, we co-expressed IE1 and Src64B in High Five cells and assessed Src64B kinase activity by monitoring its tyrosine phosphorylation. Our results showed that overexpression of IE1 significantly increased Src64B tyrosine phosphorylation compared with the EGFP control (Fig. 2A). Furthermore, we introduced a point mutation in the Y_129_FTS motif, replacing the tyrosine residue with phenylalanine. This substitution markedly reduced both IE1-Src64B binding and Src64B tyrosine phosphorylation (Fig. 2B). These results indicate that IE1 binds and activates Src64B via its Y_129_FTS tyrosine motif.

To determine whether Src kinase activity influences the interactions between IE1 and Src64B or PI3Kp85α, we performed Co-IP experiments in the presence of the specific Src family kinase inhibitor Saracatinib. Treatment with Saracatinib significantly attenuated the interaction between IE1 and Src64B or PI3Kp85α (Fig. 2C and D). Moreover, Co-IP experiments upon co-expression of IE1, Src64B, and PI3Kp85α revealed that these proteins form a ternary complex (Fig. 2E). Notably, Src64B overexpression enhanced the interaction between IE1 and PI3Kp85α (Fig. 2E), whereas Saracatinib treatment reduced these interactions (Fig. 2F). Collectively, these data demonstrate that IE1 activates Src64B, thereby facilitating the recruitment of PI3Kp85α.

### IE1 is essential for activating Src-PI3K-Akt signaling upon WSSV infection

**Fig 3:**
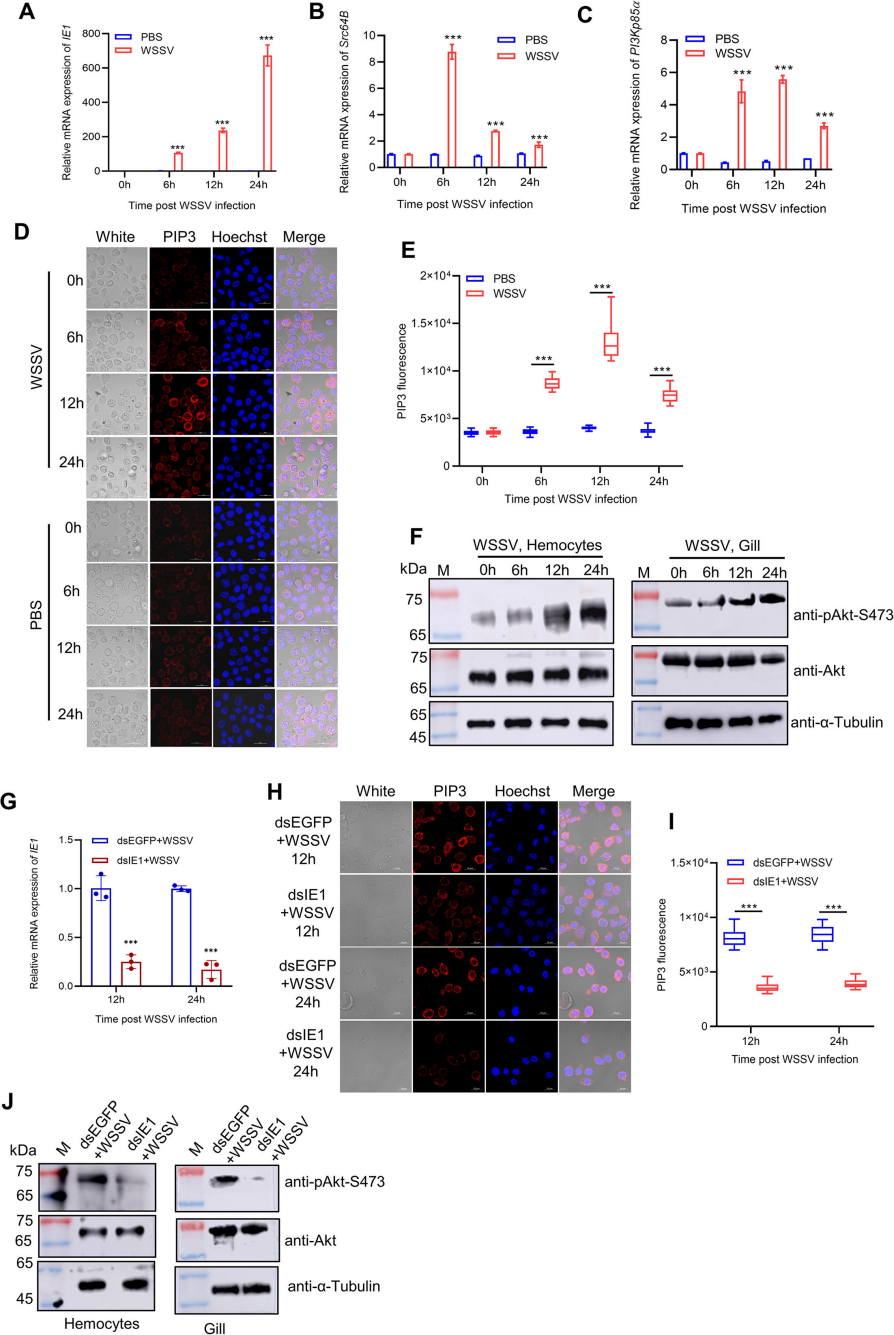
IE1 is essential for PI3K-Akt pathway activation during WSSV infection. (**A–C**) WSSV infection upregulates Src64B and PI3Kp85α expression. Hemocytes collected at 0, 6, 12, and 24 h post-infection with WSSV or PBS were analyzed by qPCR for mRNA expression of (**A**) IE1, (**B**) Src64B, and (**C**) PI3Kp85α. Data are from three independent biological replicates (*n* = 3) and are normalized to PBS controls at each time point. (**D, E**) WSSV infection stimulates PIP3 production. Hemocytes collected post-WSSV or PBS injection were used for immunofluorescence staining with an anti-PIP3 antibody. (**D**) Representative immunofluorescence images of PIP3 staining at the indicated time points. Scale bar, 10 µm. (**E**) Quantification of PIP3 fluorescence intensity (*n* = 100 hemocytes) from panel D. (**F**) Akt is activated after WSSV infection. Hemocytes and gills were collected post-WSSV injection and lysed for western blot analysis using anti-pAkt-S473 and anti-Akt antibodies. (**G–J**) Knockdown of IE1 inhibits PIP3 production and Akt activation. Shrimp were injected with dsIE1 or dsEGFP (control) followed by WSSV infection at 12 h. (**G**) qPCR analysis of IE1 knockdown efficiency in hemocytes. The results are representative of three independent experiments (*n* = 3). (**H**) Representative PIP3 staining images. Scale bar, 10 µm. (**I**) Quantified PIP3 fluorescence intensity (*n* = 100 hemocytes) from panel H. (**J**) Western blot analysis of p-Akt (Ser473) and total Akt at 24 h post-infection in hemocytes and gills. Statistical significance was determined using a two-tailed Student’s *t*-test. *** *P* < 0.001.

Previous studies have established the essential role of the PI3K-Akt pathway in promoting WSSV infection (15–17); however, the dynamics of its activation during infection remain unclear. To address this, we quantified the mRNA expression of Src64B and PI3Kp85α, as well as monitored PIP3 generation and Akt phosphorylation (pAkt-Ser473) in hemocytes at various time points post-injection with WSSV or PBS. The results showed that a progressive increase in IE1 mRNA expression was observed following WSSV injection (Fig. 3A), indicating successful viral infection. Notably, the mRNA levels of Src64B and PI3Kp85α in hemocytes were transiently upregulated at 6 h post-infection, then declined from 12 to 24 h post-infection compared with the PBS controls (Fig. 3B and C). Moreover, in both hemocytes and gills, PIP3 generation and Akt phosphorylation, which are key markers of PI3K-Akt pathway activation (10), were significantly elevated from 6 to 24 h post-WSSV infection relative to the PBS controls (Fig. 3D through F). These findings suggest that the PI3K-Akt pathway is activated during the early stage of WSSV infection.

Given that Src and PI3Kp85ɑ are two critical components of the mammalian PI3K-Akt pathway (30, 31), we investigated whether the WSSV IE1 protein regulates this pathway by interacting with *P. vannamei* Src64B and PI3Kp85α. We first examined the involvement of Src64B and PI3Kp85α in activating the PI3K-Akt pathway in shrimp during WSSV infection. Our results demonstrated that under WSSV infection, knockdown of Src64B or PI3Kp85α, or treatment of shrimp with the Src inhibitor Saracatinib or the PI3K inhibitor LY294002, significantly reduced PIP3 generation and Akt phosphorylation in hemocytes post-WSSV infection (Fig. S2A through H). These results underscore the evolutionary conservation of the PI3K-Akt pathway across species. Subsequently, we determined the role of IE1 in regulating this pathway. Knockdown of the IE1 gene in WSSV-infected shrimp resulted in a notable reduction in PIP3 generation and Akt phosphorylation in hemocytes and gills (Fig. 3G through J). Overall, our results indicate the essential role of IE1 in activating the Src-PI3K-Akt signaling upon WSSV infection.

### Src-PI3K-Akt signaling inhibits apoptosis and autophagy during WSSV infection

**Fig 4:**
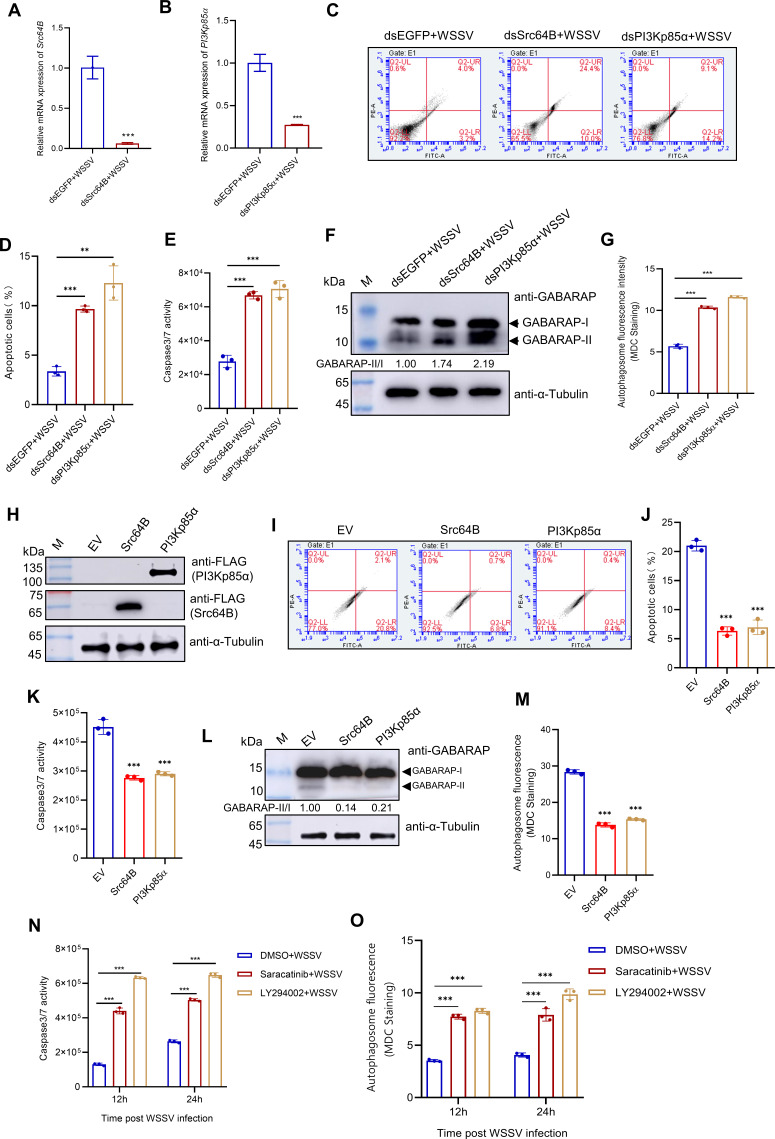
Src-PI3K-Akt signaling inhibits apoptosis and autophagy during WSSV infection. (**A–G**) Knockdown of Src64B and PI3Kp85α promotes apoptosis and autophagy. Shrimp were injected with dsEGFP (control), dsSrc64B, or dsPI3Kp85α, followed by WSSV infection. Hemocytes were collected at 24 h post-infection and used for apoptosis and autophagy assays. (**A and B**) qPCR analysis of (**A**) Src64B and (**B**) PI3Kp85α knockdown efficiency. (**C**) Apoptosis detection by YO-PRO−1/PI staining and flow cytometry. (**D**) Quantification of apoptotic rates from panel C. (**E**) Measurement of Caspase 3/7 activity. (**F**) Western blot analysis of autophagy marker GABARAP-II. (**G**) MDC staining for autophagosome visualization. (**H–M**) Overexpression of Src64B and PI3Kp85α attenuates apoptosis and autophagy. High Five cells were transfected with empty vector (EV), Src64B-FLAG, or PI3Kp85α-FLAG and then harvested at 48 h to detect apoptosis and autophagy. (**H**) Western blot analysis of Src64B and PI3Kp85α expression. (**I**) Apoptosis assessment by YO-PRO−1/PI staining and flow cytometry. (**J**) Quantified apoptotic rates from panel I. (**K**) Detection of Caspase 3/7 activity. (**L**) GABARAP-II protein levels by western blot. (**M**) MDC staining for autophagy evaluation. (**N, O**) Saracatinib and LY294002 treatment enhance apoptosis and autophagy. Shrimp were injected with WSSV plus DMSO (control), Saracatinib (Src inhibitor, 10 µM), or LY294002 (PI3K inhibitor, 20 µM), and hemocytes were then collected at 12 and 24 h post-infection for Caspase 3/7 activity (**N**) and MDC staining analyses (**O**). All data are presented as the mean ± SD from three independent biological replicates (*n* = 3). Statistical significance was determined by a two-tailed Student’s *t*-test. Significance levels are denoted as ns (not significant), ** *P* < 0.01 and *** *P* < 0.001.

To investigate the role of the Src-PI3K-Akt signaling during infection, we performed comparative transcriptomic analyses following PI3Kp85α silencing in WSSV-infected shrimp. The results revealed significant upregulation of genes associated with pro-apoptotic and autophagy pathways in PI3Kp85α-silenced shrimp (Fig. S3A and B), suggesting that the PI3K pathway may act as a negative regulator of apoptosis and autophagy during infection. We then conducted RNA interference (RNAi) and overexpression experiments targeting Src64B and PI3Kp85α to validate this hypothesis. Silencing Src64B or PI3Kp85α in WSSV-infected shrimp led to a marked increase in the percentage of apoptotic cells and Caspase activation, as demonstrated by flow cytometry and Caspase 3/7 activity assays (Fig. 4A through E). Conversely, overexpression of these proteins in High Five cells reduced apoptosis (Fig. 4H through K). Autophagy levels were assessed using western blotting and Monodansylcadaverine (MDC) staining. Silencing Src64B or PI3Kp85α in WSSV-infected shrimp significantly increased the ratio of γ-aminobutyric acid receptor-associated protein II/I (GABARAP-II/I), a marker of autophagy induction (32), and promoted autophagosome formation (Fig. 4F and G). Overexpression of these proteins, however, suppressed autophagy (Fig. 4L and M). Furthermore, inhibitor experiments showed that treating WSSV-infected shrimp with the Src inhibitor Saracatinib or the PI3K inhibitor LY294002 induced apoptotic and autophagic processes (Fig. 4N and O). These results confirm that the Src-PI3K signaling inhibits apoptosis and autophagy during WSSV infection.

To clarify whether the Src-PI3K signaling exerts its effects through its downstream effector Akt, we treated WSSV-infected shrimp with a specific Akt inhibitor MK2206. The results demonstrated that this inhibitor induced apoptosis and autophagy compared with the control group (Fig. S4A and B). Moreover, when cells overexpressing Src64B or PI3Kp85α were further treated with the Akt inhibitor, apoptosis and autophagy levels were significantly increased compared with untreated overexpressing cells (Fig. S4C through E). These results strongly indicate that the Src-PI3K-Akt signaling suppresses apoptosis and autophagy under WSSV challenge.

### IE1 inhibits apoptosis and autophagy via the Src-PI3K-Akt signaling

**Fig 5:**
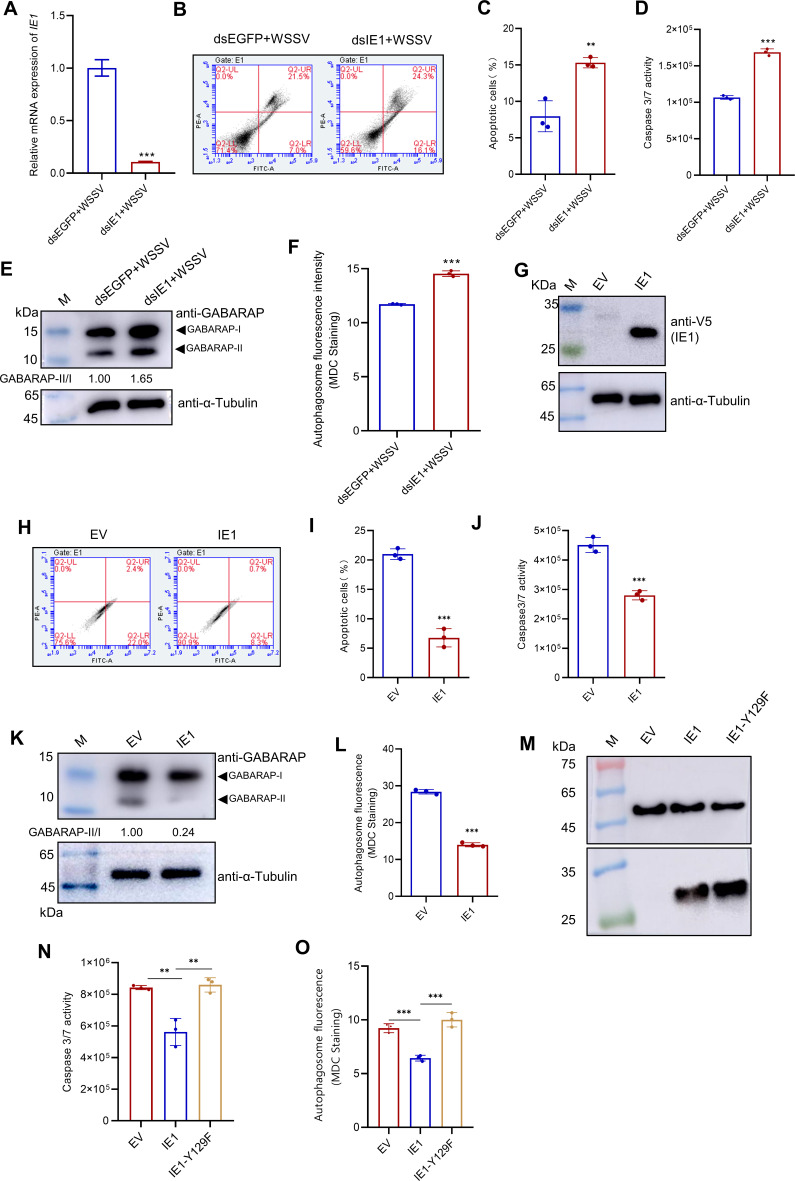
IE1 inhibits apoptosis and autophagy during WSSV infection. (**A–F**) Knockdown of IE1 promotes apoptosis and autophagy. Shrimp were injected with dsEGFP (control) or dsIE1 and then infected with WSSV at 12 h post-knockdown. Hemocytes were collected at 24 h post-infection and subjected to apoptosis and autophagy analyses. (**A**) qPCR validation of IE1 knockdown efficiency. (**B**) Apoptosis assessment by YO-PRO−1/PI staining and flow cytometry. (**C**) Quantification of apoptotic rates from panel B. (**D**) Detection of Caspase 3/7 activity. (**E**) Western blot analysis of the autophagy marker GABARAP-II. (**F**) Autophagic vacuoles visualized by MDC staining. (**G–L**) Overexpression of IE1 inhibits apoptosis and autophagy. High Five cells were transfected with empty vector (EV) or IE1-V5 plasmids. At 48 h post-transfection, apoptosis and autophagy were evaluated. (**G**) Western blot analysis of IE1 expression. (**H**) Apoptosis analysis by YO-PRO−1/PI staining and flow cytometry. (**I**) Quantified apoptotic rates from panel H. (**J**) Determination of Caspase 3/7 activity. (**K**) GABARAP-II protein levels by western blot. (**L**) Autophagy assessment via MDC staining. (**M–O**) Effect of IE1-Y129F mutant overexpression on apoptosis and autophagy. High Five cells were transfected with empty vector (EV), IE1-V5, and IE1-Y129F mutant plasmids. At 48 h post-transfection, apoptosis and autophagy were evaluated. (**M**) Western blot analysis of IE1 and IE1-Y129F expression. (**N**) Determination of Caspase 3/7 activity. (**O**) Autophagy assessment via MDC staining. All data are representative of three independent experiments (*n* = 3), and statistical significance was determined using a two-tailed Student’s *t*-test. ** *P* < 0.01 and *** *P* < 0.001.

Building on the above findings, we investigated the role of the WSSV IE1 protein in regulating apoptosis and autophagy. Using RNAi and overexpression experiments, we evaluated the effect of IE1 on these processes. In WSSV-infected shrimp, knockdown of IE1 significantly increased the percentage of apoptotic cells and enhanced caspase 3/7 activity (Fig. 5A through D) while also elevating the GABARAP II/I ratio and promoting autophagosome formation (Fig. 5E and F). Conversely, overexpression of IE1 in High Five cells suppressed both apoptosis and autophagy (Fig. 5G through L). To assess whether the Y_129_FTS tyrosine motif is required for the suppressive function of IE1 on apoptosis and autophagy, we overexpressed the IE1-Y129F mutant in High Five cells. In contrast to the wild-type IE1, the IE1-Y129F mutant failed to suppress either apoptosis or autophagy, evidenced by the observation that apoptosis and autophagy levels in cells expressing IE1-Y129F were comparable with those in empty vector controls (Fig. 5M through O). This indicates that an intact Y129 motif is essential for IE1-mediated inhibition of apoptosis and autophagy.

**Fig 6:**
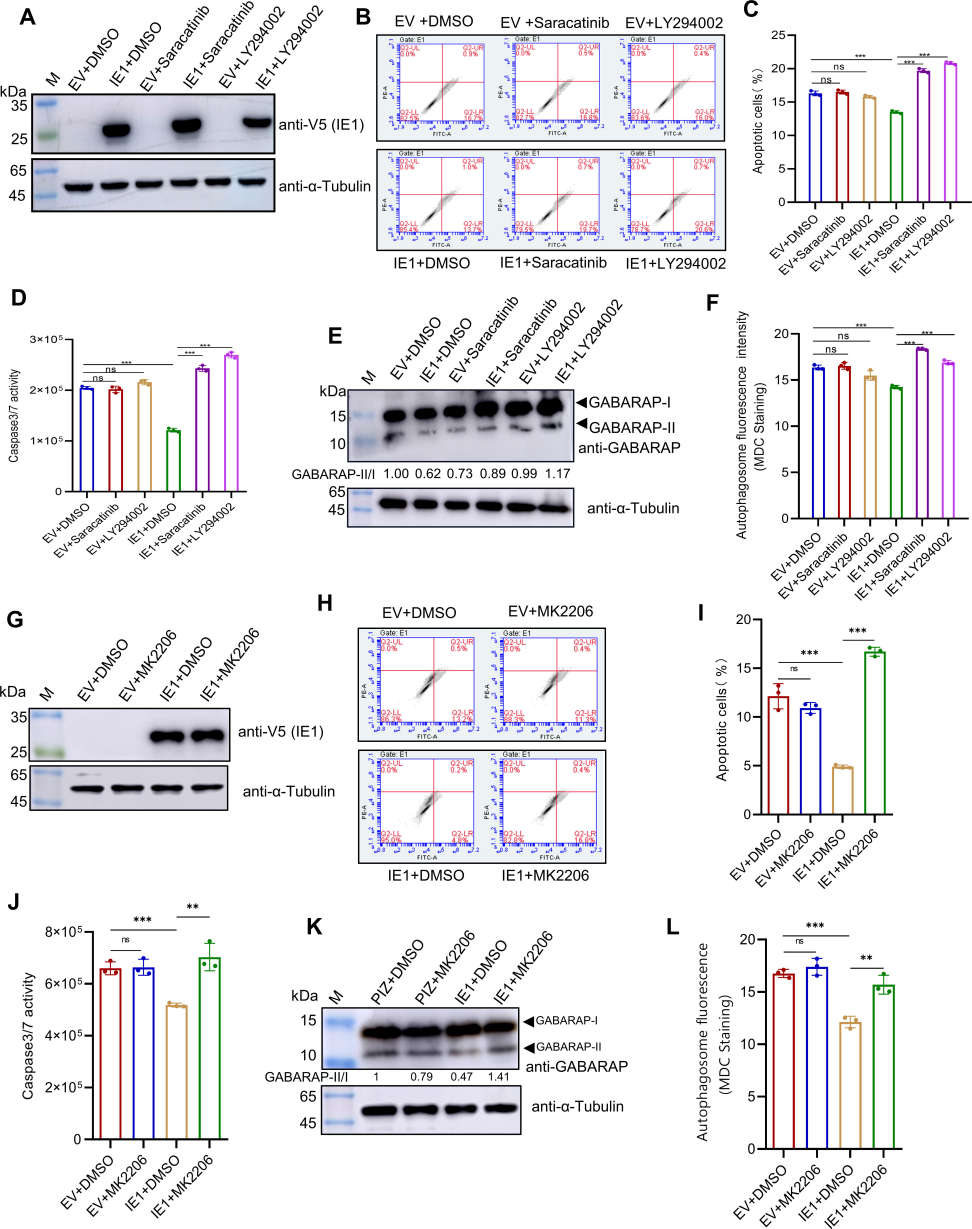
IE1 suppresses apoptosis and autophagy through Src-PI3K-Akt signaling. High Five cells were transfected with either empty vector (EV) or IE1-V5, followed by treatment with Saracatinib (Src inhibitor), LY294002 (PI3K inhibitor), MK2206 (Akt inhibitor), or DMSO (control) at 24 h post-transfection. Cells were harvested at 48 h post-transfection and used for the apoptosis and autophagy assays. (**A and G**) Western blot confirming the expression of IE1. (**B and H**) Apoptosis detection via YO-PRO−1/PI staining and flow cytometry. (**C and I**) Quantification of apoptotic rates from panel (**B and H**). (**D and J**) Detection of Caspase 3/7 activity. (**E and K**) Western blot analysis of autophagy marker GABARAP-II. (**F and L**) Autophagosome visualization using MDC staining. All data are derived from three independent biological replicates (*n* = 3). Statistical significance was assessed by a two-tailed Student’s *t*-test, with ns indicating not significant, **P < 0.01, and ***P < 0.001.

To further examine whether IE1 exerts its suppressive effect through activation of the Src-PI3K-Akt signaling pathway, we treated IE1-overexpressing cells with inhibitors targeting Src, PI3K, and Akt. The results showed that apoptosis and autophagy levels were significantly higher in inhibitor-treated cells compared with untreated controls (Fig. 6A through L). Taken together, these results demonstrate that IE1 inhibits apoptosis and autophagy via the Src-PI3K-Akt signaling pathway during WSSV infection.

### IE1-induced Src-PI3K-Akt signaling facilitates WSSV infection by suppressing apoptosis and autophagy

**Fig 7:**
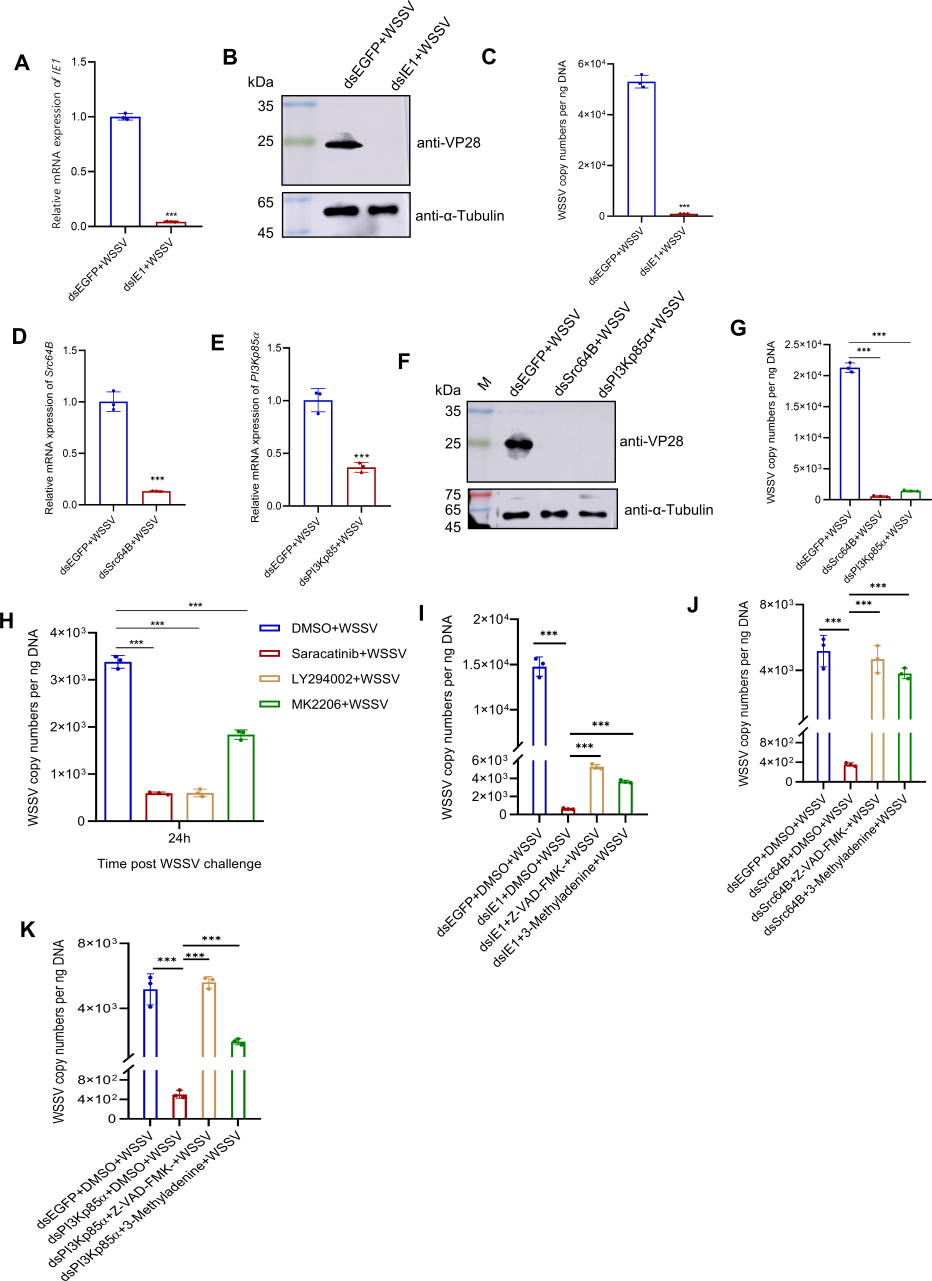
IE1-inducing Src-PI3K-Akt signaling facilitates WSSV infection by suppressing apoptosis and autophagy. (**A-G**) Knockdown of IE1, Src64B, and PI3Kp85α reduces WSSV infection. Shrimp were injected with dsEGFP (control), dsIE1, dsSrc64B, or dsPI3Kp85α, followed by WSSV infection. Hemocytes collected at 24 h post-infection were used for WSSV proliferation analysis. (**A, D, and E**) qPCR validation of IE1 (**A**), Src64B (**D**), and PI3Kp85ɑ (**E**) knockdown efficiency. (**B and F**) Western blot analysis of WSSV VP28 protein. (**C and G**) Quantification of WSSV copy number. (**H**) Pharmacological inhibition of Src-PI3K-Akt signaling suppresses WSSV infection. Shrimp were injected with WSSV plus Saracatinib (Src inhibitor), LY294002 (PI3K inhibitor), MK2206 (Akt inhibitor), or DMSO (control), and hemocytes were then collected at 24 h post-infection for WSSV copy number quantification. (**I–K**) Pharmacological inhibition of apoptosis and autophagy counteracts the suppression of WSSV infection caused by gene knockdown. Shrimp were injected with dsIE1 (**I**), dsSrc64B (**J**), or dsPI3Kp85α (**K**). At 12 h post-IE1 knockdown or 24 h post-Src64B/PI3Kp85α knockdown, shrimp were further injected with WSSV plus apoptosis or autophagy inhibitor (Z-VAD-FMK or 3-Methyladenine). Hemocytes were collected at 24 h post-infection for viral copy number quantification. All data are from three independent biological replicates (*n* = 3), and statistical significance was determined using a two-tailed Student’s *t*-test. *** *P* < 0.001.

To determine the role of the IE1-induced Src-PI3K-Akt signaling in WSSV infection, we performed individual knockdowns of IE1, Src64B, and PI3Kp85α in WSSV-infected shrimp and assessed their impact on viral infection. The results showed that silencing these genes led to a significant reduction in WSSV VP28 gene expression and viral copy numbers compared with the control groups (Fig. 7A through G), indicating the essential role of this IE1-induced signaling axis in WSSV infection. This conclusion was further supported by inhibitor studies, where treatment of WSSV-infected shrimp with inhibitors targeting Src, PI3K, or Akt also resulted in a significant decrease in WSSV copy numbers relative to untreated controls (Fig. 7H). Furthermore, to determine whether the IE1-induced Src-PI3K-Akt signaling promotes WSSV infection by inhibiting apoptosis and autophagy, we individually injected apoptosis and autophagy inhibitors (Z-VAD-FMK and 3-Methyladenine) into shrimp that had been depleted of IE1, Src64B, and PI3Kp85α and then analyzed their effect on WSSV infection. The results demonstrated that in the IE1, Src64B, and PI3Kp85α-depleted shrimp, treatment with apoptosis and autophagy inhibitors increased WSSV copy numbers compared with untreated shrimp (Fig. 7I through K). These results indicate that IE1-induced Src-PI3K-Akt signaling facilitates WSSV infection by suppressing apoptosis and autophagy.

## DISCUSSION

Previous research has demonstrated that the PI3K-Akt pathway facilitates WSSV infection by reprogramming metabolic processes, maintaining cellular redox balance, and enhancing lipid synthesis in shrimp (15–17). However, the precise mechanism through which WSSV activates this pathway has remained elusive. Here, our study identifies the viral immediate-early protein IE1 as the key trigger. We reveal that IE1 binds to and activates the host kinase Src64B via its Y_129_FTS tyrosine motif, leading to the recruitment of PI3Kp85α to the plasma membrane and initiation of the PI3K-Akt signaling cascade. This IE1-triggered activation of the Src–PI3K–Akt axis suppresses host apoptotic and autophagic responses, thereby establishing a cellular environment conducive to viral proliferation. Critically, we provide direct functional evidence linking this molecular mechanism to phenotypic outcome: the IE1-Y129F mutant, which is impaired in Src64B binding and activation, fails to suppress apoptosis and autophagy. This establishes Y129-mediated activation of the Src-PI3K-Akt pathway as the pivotal upstream event through which WSSV neutralizes these essential host defense mechanisms. The residual PIP3 and pAkt signals observed following IE1 knockdown likely reflect incomplete gene silencing and/or the contribution of alternative WSSV-mediated PI3K-Akt pathway activation mechanisms. Nonetheless, the marked reduction in pathway activation underscores the essential and direct role of IE1 in hijacking this specific signaling axis upon WSSV infection. Collectively, our findings thus offer the first mechanistic explanation of how WSSV coopts the host PI3K-Akt pathway via IE1 to achieve immune evasion.

The PI3K-Akt pathway is a core regulator of cellular homeostasis, making it a prime target for viral manipulation. Viruses such as Influenza A virus, Hepatitis C virus, and Herpes simplex virus type 1 have been shown to activate the PI3K-Akt pathway to promote their proliferation (33–35). Our data confirm that WSSV likewise activates this pathway during the early stages of infection, as evidenced by increased PIP3 generation and Akt phosphorylation during this phase. Although the outcome is conserved, the strategies employed by viruses vary. For instance, the NS1 protein of the Influenza A virus and the NS5A protein of the Hepatitis C virus directly interact with the SH3 domain of PI3Kp85 to trigger its activation (34, 36). In contrast, Newcastle disease virus utilizes a more indirect mechanism, where its V protein promotes the ubiquitin-mediated degradation of PHLPP2, thereby relieving the inhibition on Akt (37). A third, recurrent tactic involves the hijacking of Src family kinases. The tegument protein VP11/12 of Herpes simplex virus type 1 and the middle T antigen (MTAg) of polyomavirus contain tyrosine motifs that mediate binding to and activation of host Src kinases. The activated Src then phosphorylates tyrosine motifs within VP11/12 and MTAg, creating docking sites for PI3Kp85 recruitment and subsequent pathway activation (28, 29). Notably, we found that the WSSV IE1 protein employs a mechanism analogous to that of VP11/12 and MTAg: IE1 associates with Src64B via its Y_129_FTS tyrosine motif, triggering Src64B activation, PI3Kp85 recruitment, and PI3K-Akt signaling. Furthermore, our domain analysis revealed that *P. vannamei* PI3Kp85α possesses two additional C1 domains and an N-terminal SAM domain, which are absent in its human ortholog. As these domains are known to mediate protein-protein and protein-lipid interactions in other systems (38, 39), we speculate that these unique structural features may facilitate WSSV-specific interactions, potentially enhancing the recruitment of PI3Kp85α to the plasma membrane upon IE1-Src64B complex formation. This could represent an evolutionary adaptation of WSSV to its crustacean host, optimizing viral hijacking of the Src-PI3K-Akt axis. Future structural and mutational studies focusing on these domains will be essential to elucidate their precise roles in WSSV infection.

Having established how IE1 activates the pathway, we sought to define the specific downstream processes it modulates to support viral proliferation. Transcriptomic profiling following PI3Kp85α knockdown in WSSV-infected shrimp revealed a significant upregulation of apoptosis- and autophagy-related genes, suggesting that IE1-driven signaling acts to suppress both processes. This finding is highly relevant, as apoptosis and autophagy are well-established host defense mechanisms that restrict viral propagation (40, 41). Their inhibition is a common viral evasion strategy, particularly critical in invertebrates like shrimp, which lack a vertebrate-like adaptive immune system and rely heavily on these innate cellular defenses. Indeed, WSSV has evolved a repertoire of strategies to counteract these responses. To inhibit apoptosis, it encodes proteins such as the E3 ligase WSSV222, which mediates ubiquitination and degradation of a host tumor suppressor-like protein (42); AAP-1 (WSSV449), which binds and inhibits the effector caspase PmCasp (43), and other anti-apoptotic factors, including WSSV134, WSSV322, and ORF390, which suppress caspase-dependent apoptosis (44, 45). Additionally, WSSV employs a viral miRNA, WSSV-miR-N24, to post-transcriptionally silence host caspase 8 and repress apoptosis (46). In parallel, WSSV subverts autophagy through the tegument protein VP26, which binds SNAP29 and disrupts SNARE complex assembly, thereby blocking autophagosome-lysosome fusion and autophagic flux (47). Our present study adds a novel and overarching layer to this understanding by revealing that WSSV, through its IE1 protein, actively suppresses both apoptosis and autophagy simultaneously by hijacking the host Src-PI3K-Akt signaling pathway. This mechanism is consistent with the established role of PI3K-Akt signaling in facilitating viral replication across diverse viruses, such as Influenza A virus and Newcastle disease virus, which activate this pathway for antiapoptotic effects (37, 48), and Human papillomavirus type 16 and Rotavirus, which exploit it to inhibit autophagy (49, 50).

Furthermore, our discovery of IE1 as the viral trigger of the Src-PI3K-Akt axis provides a potential mechanistic explanation for the previously reported WSSV-induced metabolic reprogramming. Earlier studies have established that WSSV infection hijacks the PI3K-Akt pathway to induce aerobic glycolysis (the Warburg effect) and lipid biosynthesis, processes crucial for viral replication (15–17). Our demonstration that IE1 is the key viral factor responsible for the early activation of this pathway makes it plausible that the IE1-Src-PI3K-Akt signaling cascade we delineated serves as the upstream driver of these metabolic alterations. Although the observed increase in apoptotic and autophagic cells following Src-PI3K pathway disruption may appear numerically limited, it is biologically significant in the context of viral infection, where even a partial attenuation of pro-survival signaling can substantially impair viral yield. Moreover, this effect represents only one facet of the pathway’s role. We propose that by initiating this pathway, IE1 not only directly suppresses apoptosis and autophagy but also orchestrates a broader pro-viral state, including metabolic reprogramming. This integrated model suggests that WSSV, through a single viral protein, co-opts a central host signaling hub to achieve multi-faceted immune evasion: simultaneously blocking key cellular defense pathways and reprogramming cellular metabolism to fuel its proliferation. Future research aimed at directly linking IE1 expression to specific metabolic changes will be valuable in fully elucidating this coordinated evasion strategy.

**Fig 8:**
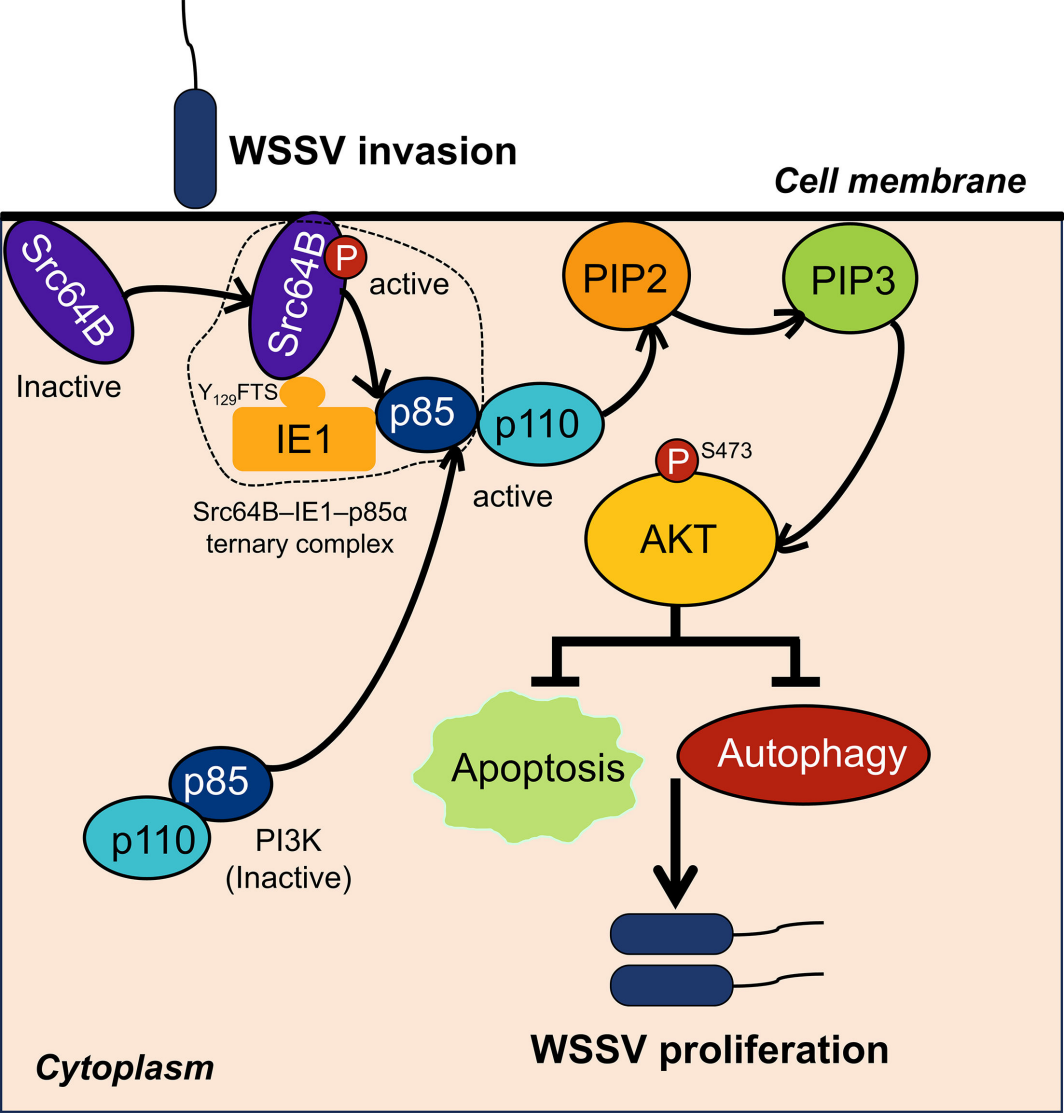
Mechanism diagram of WSSV IE1 protein hijacking host Src-PI3K-Akt signaling to facilitate viral proliferation. Our results demonstrate that the WSSV-encoded IE1 protein hijacks host cellular machinery by binding to and activating Src64B kinase via its Y_129_FTS tyrosine motif, leading to recruitment of PI3Kp85α and subsequent activation of the PI3K-Akt signaling cascade. This pathway activation suppresses host apoptotic and autophagic responses, facilitating viral proliferation.

In summary, our study reveals a novel mechanism by which WSSV IE1 co-opts host Src-PI3K-Akt signaling to inhibit apoptosis and autophagy, thereby promoting viral proliferation (Fig. 8). These findings establish the Src-PI3K-Akt axis as a critical determinant of WSSV pathogenesis and identify its components as promising therapeutic targets. Although this work defines the role of IE1 in initiating this signaling cascade, several questions remain. In particular, the structural basis for the IE1-Src64B interaction warrants further investigation. Based on the canonical activation mechanism of Src family kinases and our mutational analysis (28, 35), we hypothesize that the phosphorylated Y_129_FTS motif of IE1 binds to the SH2 domain of Src64B, thereby disrupting its auto-inhibitory conformation and leading to kinase activation. Future studies employing techniques such as X-ray crystallography or cryo-EM to resolve the complex structure of IE1 bound to Src64B will be crucial to validate this hypothesis and clarify the precise molecular interfaces involved. Additionally, the potential contribution of other WSSV proteins to pathway subversion cannot be excluded, given that viruses often employ redundant mechanisms to ensure host manipulation.

## MATERIALS AND METHODS

### Experimental animals

Pacific white shrimp (*P. vannamei*), 5–8 g body weight, were obtained from a local shrimp farm in Shantou, Guangdong, China. Prior to experimentation, the animals were acclimated for at least 48 h in aerated, recirculating artificial seawater (salinity 5 ‰, 25°C) and fed a commercial diet once daily.

### Antibodies, inhibitors, and plasmids

The primary antibodies used in the study included mouse anti-FLAG antibody (Beyotime, China; Cat. No. AF519), mouse anti-V5 antibody (TransGen Biotech, China; Cat. No. HT401-01), rabbit anti-phosphotyrosine antibody (Blue Light, China; Cat. No. ICP9805), mouse anti-α-tubulin antibody (Sigma, USA; Cat. No. T5168), rabbit anti-p-Akt-S473 antibody (ABclonal, China; Cat. No. AP1208), rabbit anti-Akt antibody (Servicebio, China; Cat. No. GB15689-100), and rabbit anti-GABARAP antibody (Abcam, UK; Cat. No. ab109299), and mouse anti-PIP3 antibody (ThermoFisher Scientific; USA, Cat. No. A-21328). The mouse anti-VP28 antibody was provided by Prof. Shengkang Li’s research group at Shantou University. For western blot analysis, secondary antibodies used were goat anti-mouse IgG-HRP (ThermoFisher Scientific, USA, Cat. No. G21040) and goat anti-rabbit IgG-HRP (ThermoFisher Scientific, USA; Cat. No. G21234). For the immunofluorescence assay, secondary antibodies included donkey anti-rabbit IgG-Alexa Fluor 555 (Beyotime, China; Cat. No. A0453) and goat anti-mouse IgG-Alexa Fluor 488 (Beyotime, China; Cat. No. A0428). The inhibitors used were Saracatinib (Beyotime, China; Cat. No. SC1040), LY294002 (Beyotime, China; Cat. No. S1737), MK2206 (Beyotime, China; Cat. No. SF2712), Z-VAD-FMK (Beyotime, China; Cat. No. C1202), and 3-Methyladenine (MedChemExpress, China; Cat. No. HY-19312).

The V5-tagged IE1 and EGFP expression plasmids (IE1-V5 and EGFP-V5) were constructed in our previous study (26). The IE1 Y_129_FTS tyrosine motif mutant (IE1-Y129F-V5) was generated by overlap PCR and cloned into the pIZ-V5-His vector (Invitrogen, USA). For Src64B (GenBank accession No. MH397363.1) and PI3Kp85α (GenBank accession No. MH397365.1) expression plasmids, the full-length open reading frames (ORFs) were amplified and fused with a FLAG tag and then ligated into the pIZ-V5-His and pIEx-4 (Novagen, USA) vectors, respectively. All primers used for plasmid construction are listed in Table S1.

### Co-immunoprecipitation (co-IP) assay

High Five cells were seeded into six-well culture plates at a density of ~1 × 10⁶ cells/well and maintained in Express Five SFM medium (ThermoFisher Scientific, USA; Cat. No. 10486025) overnight. For DNA transfection, 1 µg each of FLAG-tagged Src64B and PI3Kp85α expression plasmids (Src64B-FLAG and PI3Kp85ɑ-FLAG) were co-transfected with 1 µg of either IE1-V5, IE1-Y129F-V5, or EGFP-V5 (control) using FuGENE HD transfection reagent (Promega, USA; Cat. No. E2311) according to the manufacturer’s protocol. At 48 h post-transfection, the cells were harvested and lysed with 200 µL of western and IP cell lysis buffer (Beyotime, China; Cat. No. P0013) for 20 min on ice, followed by centrifugation at 16,000 × *g* for 10 min at 4°C to obtain the supernatant. A 20 µL aliquot of the supernatant was reserved for direct western blot analysis, whereas the rest was incubated with 5 µL of anti-FLAG M2 magnetic beads (Sigma, USA; Cat. No. A2220) overnight at 4°C; the beads were then washed three times with cell lysis buffer and resuspended in 20 µL PBS plus 5 µL 5 × loading buffer before boiling at 100°C for 10 min for subsequent western blot analysis.

### Immunofluorescence assay

High Five cells seeded in six-well plates were transfected with 1 µg of plasmids encoding either IE1, Src64B, or PI3Kp85α individually, or co-transfected with IE1 plus Src64B or PI3Kp85α. After 48 h, the cells were harvested and transferred to confocal dishes for 1 h to allow adherence. Following attachment, cells were fixed with 4% paraformaldehyde for 15 min, permeabilized with 0.5% Triton X-100 in PBS for 20 min, and blocked with 3% BSA in PBS for 1 h at room temperature. Primary antibodies (mouse anti-V5 and rabbit anti-FLAG, 1:200 in 3% BSA) were incubated overnight at 4°C, followed by secondary antibody staining using Alexa Fluor 488-conjugated goat anti-mouse IgG and Alexa Fluor 555-conjugated donkey anti-rabbit IgG (both 1:400) for 1 h at room temperature. Nuclei were counterstained with Hoechst 33342 (Beyotime, China; Cat. No. C1022) before imaging with a Zeiss LSM confocal microscope.

### Detection of Src64B phosphorylation by western blot analysis

Due to the unavailability of specific phospho-antibodies against *P. vannamei* Src64B, we used a commercial phosphotyrosine antibody to detect Src64B phosphorylation *in vitro*. Briefly, High Five cells were co-transfected with the Src64B-FLAG construct along with either IE1-V5, IE1-Y129F-V5, or EGFP-V5 (control) for 48 h before harvesting. Following cell lysis and co-IP using anti-FLAG M2 magnetic beads as described above, the immunoprecipitated protein samples were resolved by 10% SDS-PAGE and transferred to PVDF membranes (Millipore, USA, Cat. No. IPVH00010) using a Mini Transblot wet transfer system (Bio-Rad Laboratories, USA). After blocking with 5% skimmed BSA in TBST (20 mM Tris, 150 mM NaCl, 0.1% Tween 20, pH 7.6) for 2 h at room temperature, membranes were probed with phosphotyrosine antibody overnight at 4°C. Following three TBST washes, membranes were incubated with secondary antibodies for 1 h at room temperature, washed again, and developed using ECL reagent (Millipore, USA, Cat. No. 203453), with signal detection performed on an Amersham Imager 600 (GE Healthcare).

### WSSV challenge, qPCR, western blot, and PIP3 level measurement

The WSSV stock was purified from WSSV-infected crayfish (*Procambarus clarkii*) and quantified by absolute qPCR as described previously (51). For the challenge experiment, shrimp were intramuscularly injected with 100 µL of WSSV virions (1 × 10^5^ copies) using a sterile syringe with a 22-gauge needle, whereas control shrimp received an equal volume of sterile PBS. At 0, 6, 12, and 24 h post-infection, hemolymph was collected from each group into 600 µL of ice-cold anticoagulant solution (258 mM Sodium citrate dihydrate, 328 mM Sodium citrate, 110 mM glucose, 140 mM NaCl, pH 6.0) using a sterile syringe. The samples were immediately centrifuged at 800 × *g* for 10 min at 4°C to pellet the hemocytes for subsequent qPCR, western blot, and PIP3 level measurement analyses.

To determine the expression of Src64B, PI3Kp85ɑ, and IE1 following WSSV infection, the total RNA was extracted from hemocytes using an RNA rapid extraction kit (Fastagen, China; Cat. No. 220010) and reverse-transcribed into cDNA with a cDNA synthesis kit (TransGen Biotech, China; Cat. No. AT311-02). qPCR was performed using a reaction mixture containing 5 µL of 2 × RealStar Green Power Mix (GenStar, Beijing, China; Cat. No. A311), 1 µL each of forward and reverse primers, 1 µL of cDNA template, and 3 µL of nuclease-free water. Amplification was carried out on a LightCycler 480 (Roche, Switzerland) under the following conditions: 95°C for 10 min (initial denaturation), followed by 45 cycles of 95°C for 15 s and 60°C for 30 s. The relative mRNA expression levels, normalized to elongation factor 1-α (EF-1α), were calculated using the 2⁻^ΔΔCT^ method (all primers are listed in Table S1). For protein analysis, hemocyte lysates were subjected to western blot analysis using antibodies against Akt, including anti-phospho-Akt (Ser473) and total anti-Akt. To assess PIP3 level, hemocytes were resuspended in Insect-XPRESS medium (Lonza, Switzerland, Cat. No.12-730Q), plated on confocal dishes at 1 × 10⁵ cells/dish, and incubated for 1 h at 28°C before immunofluorescence staining with an anti-PIP3 antibody.

### Knockdown and overexpression of IE1, Src64B, and PI3Kp85α

The double-stranded RNAs (dsRNA) targeting IE1 (dsIE1), Src64B (dsSrc64B), and PI3Kp85α (dsPI3Kp85α) were synthesized *in vitro* using the HiScribe Quick High Yield RNA Synthesis Kit (New England Biolabs, USA; Cat. No. E2050S) according to the manufacturer’s protocol, with dsEGFP serving as a negative control. For IE1 knockdown, shrimp were intramuscularly injected with 15 µg of dsIE1, followed by WSSV challenge (1 × 10⁵ copies) at 12 h post-injection. For Src64B and PI3Kp85α knockdown, shrimp received 10 µg of respective dsRNA, with WSSV inoculation (1 × 10^5^ copies) performed 24 h later. Hemocytes were collected at 24 h post-infection for subsequent analyses, including Akt phosphorylation assessment, PIP3 level quantification, and evaluation of cell apoptosis, autophagy, and WSSV copy number.

Due to the lack of shrimp cell lines, overexpression studies were conducted using High Five cells. Cells were transfected with 2 µg of expression plasmids (IE1-V5, Src64B-FLAG, or PI3Kp85α-FLAG) using FuGENE HD transfection reagent according to the manufacturer’s instructions. At 48 h post-transfection, the cells were harvested by centrifugation for downstream analyses of Akt phosphorylation, PIP3 level, apoptosis, and autophagy.

### Cell apoptosis assay

Apoptotic activity was assessed using a Dead Cell Apoptosis Kit with YO-PRO−1 and propidium iodide (PI) (Invitrogen, USA; Cat. No. V23201). Briefly, the cells were harvested, washed twice with cold PBS, and resuspended at a density of approximately 1 × 10⁶ cells/mL. For staining, 1 µL of YO-PRO−1 and 1 µL of PI were added per 1 mL of cell suspension, followed by incubation on ice for 20 min in the dark. Stained cells were then analyzed immediately using a flow cytometer (Accuri C6 Plus, BD Biosciences, USA). A minimum of 10,000 events was acquired per sample. Apoptotic cells (YO-PRO−1 positive, PI negative) were distinguished from live (double negative) and necrotic/late apoptotic (PI positive) cells, and the data were analyzed using FlowJo software (BD Biosciences). Meanwhile, Caspase-3/7 activity was measured using the Caspase-Glo 3/7 Assay System (Promega, USA; Cat. No. G8091). After cell counting with a hemocytometer, 1 × 10⁶ cells were aliquoted into 1.5 mL microcentrifuge tubes. An equal volume of Caspase-Glo3/7 reagent was added to each sample, followed by incubation at room temperature for 1 h in the dark with gentle shaking. Luminescence was quantified using a GloMax-Multi Detection System (Promega, USA).

### Cell autophagy assay

Autophagic activity was detected using Monodansylcadaverine (MDC) staining (Beyotime, China; Cat. No. C3018S) according to the manufacturer’s protocol. In brief, cells were washed with 1 × wash buffer and incubated with 0.05 mM MDC dye-loading solution for 1 h at 37°C in the dark. After incubation, cells were washed twice with 1 × wash buffer. The mean fluorescence intensity per cell of autophagic vesicles was measured using a fluorescence microplate reader (Multiskan FC, Thermo Scientific) with excitation at 335 nm and emission at 512 nm. In parallel, autophagy induction was evaluated by western blot analysis using an anti-GABARAP antibody to monitor the conversion of GABARAP-I (cytosolic form) to GABARAP-II (lipidated form bound to autophagosomes).

### Quantification of WSSV copy number

WSSV copy numbers were quantified using absolute qPCR as described previously (52). Briefly, genomic DNA (gDNA) was extracted from shrimp hemocytes using the TIANamp Marine Animals DNA Kit (TIANGEN, Beijing, China) as per the manufacturer’s instructions. The viral VP28 gene was cloned into the pMD19-T vector, and the copy number of the plasmid was determined based on its concentration and molecular weight. Standard samples were created by serially diluting the VP28 gene-containing plasmid. qPCR assays were then performed using qVP28-F/R primers (Table S1) with both standard dilutions and test gDNA samples, where Ct values from the standard samples were used to generate a standard curve, and viral copy numbers in test samples were calculated by comparing their Ct values with the standard curve.

### Inhibitor treatment assay

For overexpression studies *in vitro*, High Five cells were transfected with IE1-V5, Src64B-FLAG, or PI3Kp85-FLAG expression constructs, followed by treatment with pathway inhibitors (20 µM Saracatinib for Src inhibition, 20 µM LY294002 for PI3K inhibition, or 250 nM MK2206 for Akt inhibition). After 24 h of inhibitor treatment, cells were harvested by centrifugation for subsequent apoptosis and autophagy analyses. *In vivo* experiments, shrimp were injected with WSSV (1 × 10⁵ copies) along with the respective pathway inhibitors. Hemocytes were collected at 24 h post-infection for apoptosis, autophagy, and viral copy number quantification. In a parallel experiment, shrimp were injected with either control dsEGFP or target-specific dsRNAs (dsIE1, dsSrc64B, or dsPI3Kp85α). At 12 h for IE1 knockdown or 24 h for Src64B/PI3Kp85α knockdown, the animals were challenged with WSSV (1 × 10⁵ copies) and co-treated with either pharmacological inhibitors (20 µM Z-VAD-FMK [apoptosis inhibitor] or 2.5 mM 3-Methyladenine [autophagy inhibitor]) or vehicle control (DMSO). Hemocytes were harvested at 24 hpi for viral copy number quantification. The cytotoxicity of all inhibitors used in both High Five cells and shrimp was evaluated using a CCK-8 kit (Beyotime, China, Cat. No. C0037) according to the manufacturer’s instructions. The results confirmed that the applied concentrations did not induce significant cytotoxicity under our experimental conditions (Fig. S5)

### Statistical analysis

All data represent mean ± standard deviation (SD) from at least three independent biological replicates. Statistical comparisons were performed using two-tailed Student’s *t*-tests. Data visualizations were conducted using GraphPad Prism 9.0 (GraphPad Software, USA).

## Data Availability

All data are available in the main text and the supplemental material.

## SUPPLEMENTAL MATERIAL

Supplemental material jvi.01676-25-s0001.pdf Figures S1 to S5; Table S1.

ASM does not own the copyrights to Supplemental Material that may be linked to, or accessed through, an article. The authors have granted ASM a non-exclusive, world-wide license to publish the Supplemental Material files. Please contact the corresponding author directly for reuse.
