# Supplementary material for: Hijacking of host Src-PI3K-Akt signaling by WSSV IE1 protein suppresses apoptotic and autophagic defenses to facilitate viral proliferation
Source: J Virol. 2025 Dec 16;100(1):e01676-25. doi: 10.1128/jvi.01676-25 (PMC12817958; doi:10.1128/jvi.01676-25)
Supplement: Supplemental material — Figures S1 to S5; Table S1. [file jvi.01676-25-s0001.pdf]

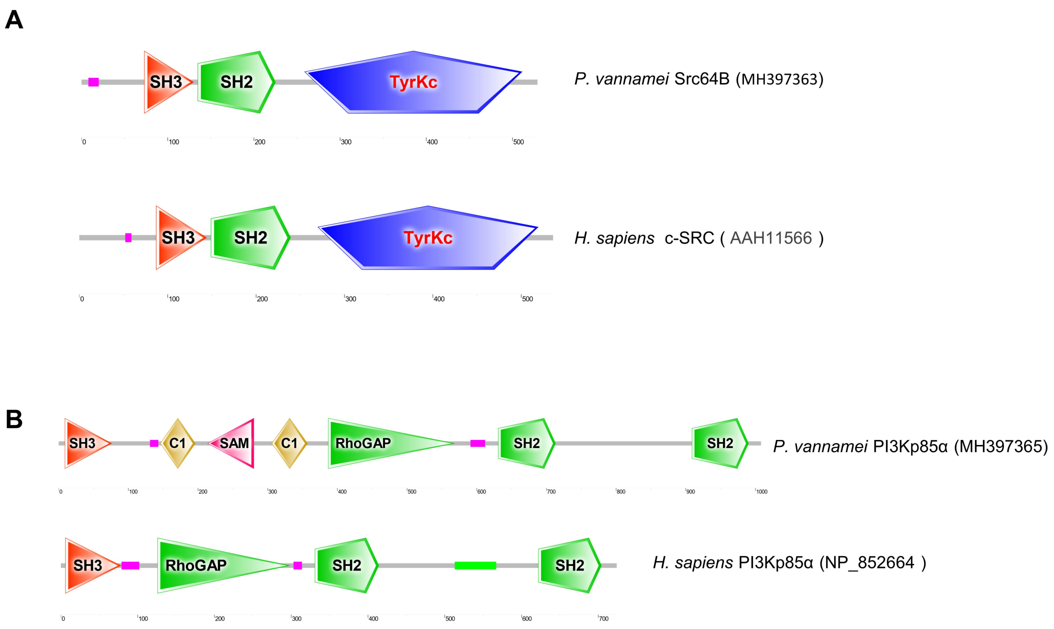

Fig. S1. Domain architecture comparison of *Penaeus vannamei* Src64B and PI3Kp85α relative to their Human orthologs. (A) Domain organization of *P. vannamei* Src64B and H. sapiens c-SRC. (B) Domain architecture of *P. vannamei* PI3Kp85α and H. sapiens PI3Kp85α.

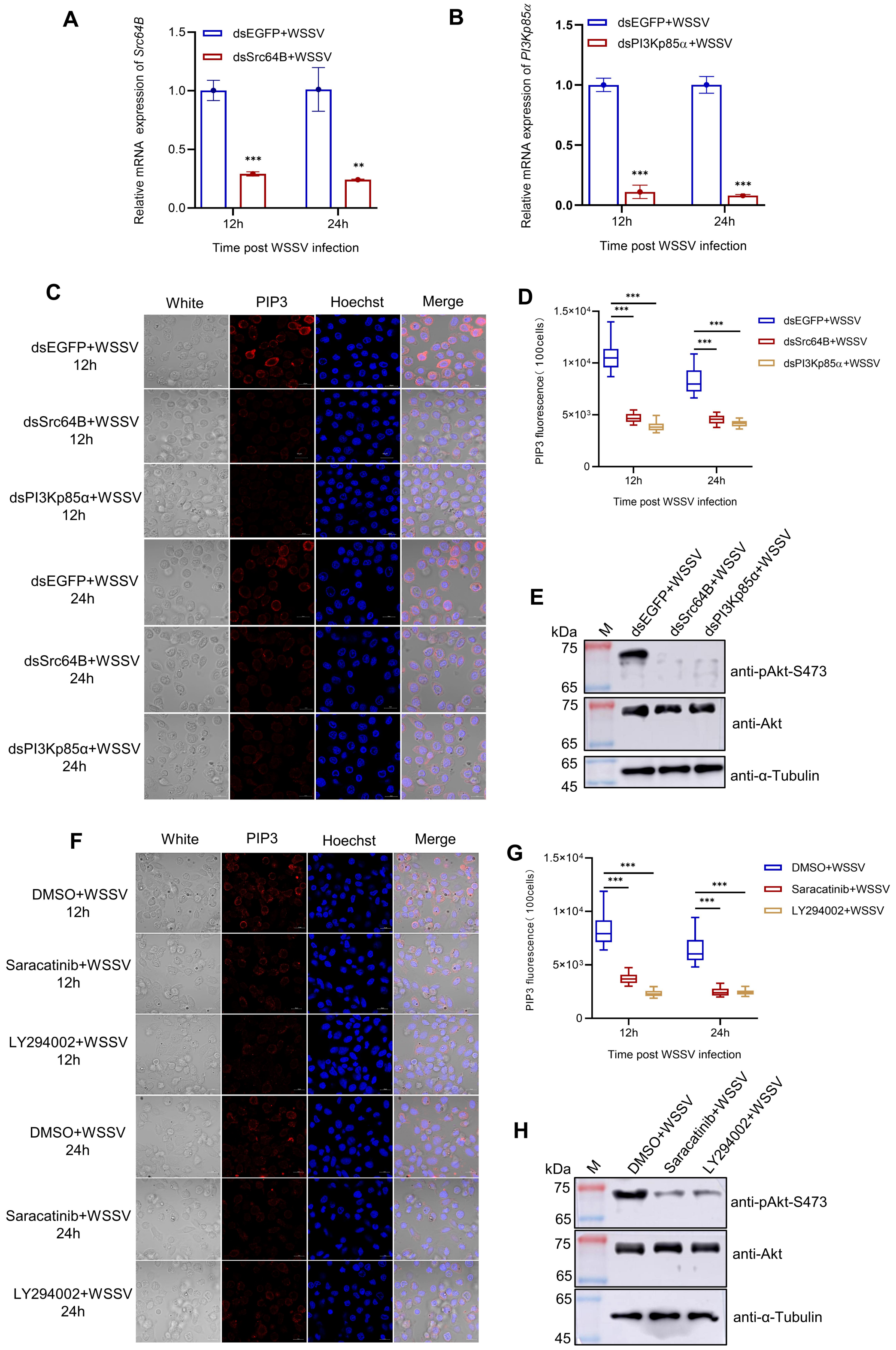

A
Relative mRNA expression of Src64B
dsEGFP+WSSV
dsSrc64B+WSSV
12h
24h
Time post WSSV infection
B
Relative mRNA expression of PI3Kp85α
dsEGFP+WSSV
dsPI3Kp85α+WSSV
Time post WSSV infection
C
White
PIP3
Hoechst
Merge
dsEGFP+WSSV 12h
dsSrc64B+WSSV 12h
dsPI3Kp85α+WSSV 12h
dsEGFP+WSSV 24h
dsSrc64B+WSSV 24h
dsPI3Kp85α+WSSV 24h
D
PIP3 fluorescence（100cells）
dsEGFP+WSSV
dsSrc64B+WSSV
dsPI3Kp85α+WSSV
Time post WSSV infection
E
kDa
M
dsEGFP+WSSV
dsSrc64B+WSSV
dsPI3Kp85α+WSSV
anti-pAkt-S473
anti-Akt
anti-α-Tubulin
F
White
PIP3
Hoechst
Merge
DMSO+WSSV 12h
Saracatinib+WSSV 12h
LY294002+WSSV 12h
DMSO+WSSV 24h
Saracatinib+WSSV 24h
LY294002+WSSV 24h
G
PIP3 fluorescence（100cells）
DMSO+WSSV
Saracatinib+WSSV
LY294002+WSSV
Time post WSSV infection
H
kDa
M
DMSO+WSSV
Saracatinib+WSSV
LY294002+WSSV
anti-pAkt-S473
anti-Akt
anti-α-Tubulin

Fig. S2. Src64B and PI3Kp85α are required for PI3K-Akt pathway activation during WSSV infection. (A-E) Knockdown of Src64B and PI3Kp85α attenuates PIP3 production and Akt activation. Shrimps were injected with dsEGFP, dsSrc64B, or dsPI3Kp85α, and then infected with WSSV at 24 h post knockdown. Hemocytes were harvested at 12 h and 24 h post-infection and used for PIP3 and Akt phosphorylation measurement. (A and B) qPCR analysis of (A) Src64B and (B) PI3Kp85α knockdown efficiency. Data are from three independent biological replicates (n = 3), and are normalized to dsEGFP controls at each time point. (C) Representative immunofluorescence images of PIP3 staining following gene knockdown. Scale bar, 10 μm. (D) Quantification of PIP3 fluorescence intensity (n=100 hemocytes) from panel C. (E) Western blot analysis of pAkt-S473 and total Akt post gene silencing. (F-H) Pharmacological inhibition of Src and PI3K suppresses PIP3 production and Akt activation. Shrimp were injected with WSSV plus Saracatinib (Src inhibitor), LY294002 (PI3K inhibitor), or DMSO (control), and hemocytes were then collected at 12 and 24 h post-infection for detecting PIP3 production and Akt phosphorylation. (F) Representative immunofluorescence images of PIP3 staining post inhibitor treatment. Scar bar, 10 μm. (G) Quantification of PIP3 fluorescence intensity (n=100 hemocytes) from panel F. (H) Western blot analysis of pAkt-S473 and total Akt after inhibitor treatment. Statistical significance was determined using a two-tailed Student's t-test. ** $P < 0.01$ and *** $P < 0.001$.

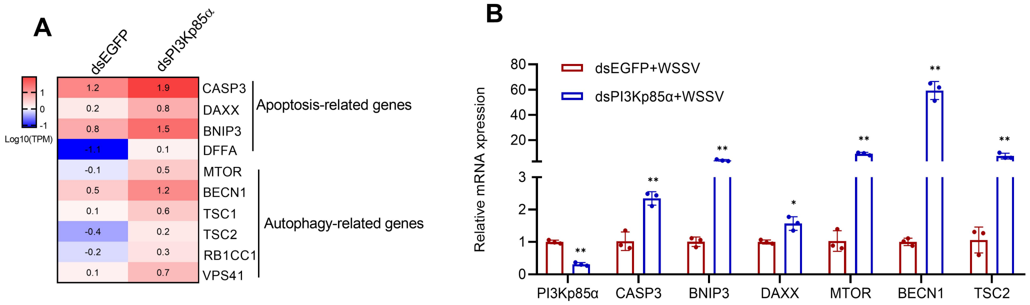

Fig. S3. Transcriptomic analysis of PI3Kp85α knockdown following WSSV infection. Shrimp were injected with dsEGFP (control) or dsPI3Kp85α, followed by WSSV infection at 48 h post dsRNA injection. At 24 h post-infection, hemocytes were collected and subjected to transcriptome sequencing analysis. (A) Heatmap showing the differential expression of apoptosis- and autophagy-related genes post PI3Kp85α knockdown. The color bar indicates the gradient of Log10-transformed normalized expression levels (TPM). (B) Validation analysis of apoptosis- and autophagy-related genes by qPCR. Results are representative of three independent biological replicates (n = 3), and statistical significance was determined using a two-tailed Student's t-test. * P <0.05, ** $P < 0.01$ and *** $P < 0.001$.

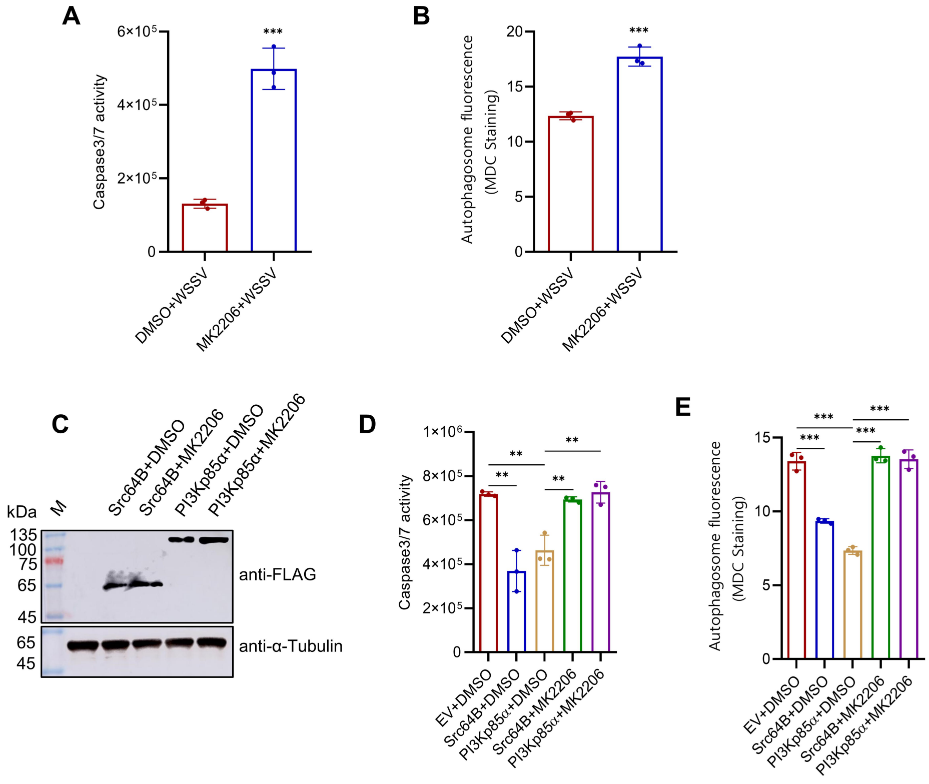

Fig. S4. Src-PI3K signaling inhibits apoptosis and autophagy via Akt. (A and B) Pharmacological inhibition of Akt suppresses apoptosis and autophagy. Shrimp were injected with WSSV plus MK2206 (Akt inhibitor), or DMSO (control), and hemocytes were then collected at 24 h post-infection for evaluation of apoptotic activity through Caspase 3/7 assay (A) and autophagic activity via MDC staining (B). (C-E) Pharmacological inhibition of Akt counteracts the suppression of apoptosis and autophagy caused by Src64B and PI3Kp85α overexpression. Results are representative of three independent biological replicates (n = 3), and statistical significance was determined using a two-tailed Student's t-test. ** $P < 0.01$ and *** $P < 0.001$.

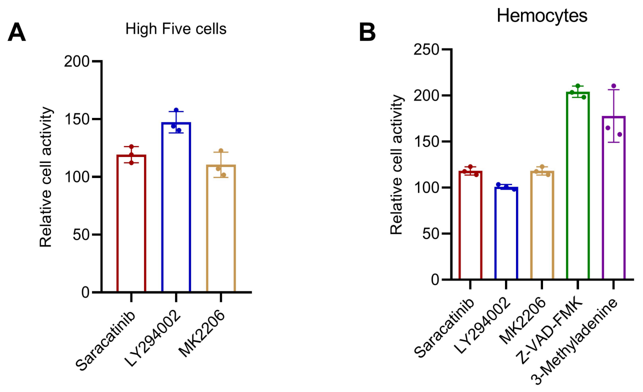

Fig. S5 Cytotoxicity assay of inhibitors employed in this study. (A) High Five cells were treated with inhibitors (20 μM Saracatinib, 20 μM LY294002, or 250 nM MK2206) for 24 h, and cell viability was assessed by CCK-8 assay. (B) Shrimp were injected with inhibitors (20 μM Saracatinib, 20 μM LY294002, 250 nM MK2206, 20 μM Z-VAD-FMK, or 2.5 mM 3-Methyladenine) for 24 h. Hemocytes were then collected for cell viability analysis.

Table S1 Primers used in this study

Primer name	Sequence (5’-3’)
**For qPCR assay**	
qEF1α-F	TATGCTCCTTTTGGACGTTTTGC
qEF1α-R	CCTTTTCTGCGGCCTTGGTAG
qIE1-F	GCACAACAACAGACCCTACCC
qIE1-R	GAAATACGACATAGCACCTCCAC
qSrc64B-F	TAAGTATAAGGGATTGGGAC
qSrc64B-R	CCTGTGATGAGGTGATGAAG
qPI3Kp85α-F	AGTATGGGAGAAGAGGGT
qPI3Kp85α-R	AGATGGGATGTTTGAGTG
qVP28-F	AAACCTCCGCATTCCTGTGA
qVP28-R	TCCGCATCTTCTTCCTTCAT
qCASP3-F	TACAAACAGATTGGAGCG
qCASP3-R	GGACAGACAGTATGAGGC
qBNIP3-F	GCCTCCAAACAGTCCTAA
qBNIP3-R	CTGCTCCAATCCCAAAT
qDAXX-F	AGTGAAGAAAGTGACCAAGCAGAGT
qDAXX-R	TCTCCCCACATCACGGAATATT
qMTOR-F	CCACCAGCGAGAATAAAA
qMTOR-R	GGGATAACCTGAGCCAAG
qBECN1-F	TCCCATCTAAATGAACAC
qBECN1-R	TATAAACCAGTCGCTACC
qTSC2-F	TGCCGACTTCCTTTCAG
qTSC2-R	TGTCTCCCTTTCCCCTA
**For RNAi assay**	
dsIE1-F	GAAGACTCTACAAATCTCTTT
dsIE1 -R	CTTGCACCTACACGCATTACA

dsIE1-T7-F	GGATCCTAATACGACTCACTATAGGGAAGACTCTACAAATCTCTTT
dsIE1-T7-R	GGATCCTAATACGACTCACTATAGGCTTGCACCTACACGCATTACA
dsSrc64B-F	TCCTTTGGTGAAGTGTGG
dsSrc64B-R	ATTGAGTTTGAGGGTGCG
dsSrc64B-T7-F	GGATCCTAATACGACTCACTATAGGTCCTTTGGTGAAGTGTGG
dsSrc64B-T7-R	GGATCCTAATACGACTCACTATAGGATTGAGTTTGAGGGTGCG
dsPI3Kp85α-F	CTGATTGACCGCCTAGAAC
dsPI3Kp85α-R	GCAAAACCATAGCCATGTT
dsPI3Kp85α-T7-F	GGATCCTAATACGACTCACTATAGGCTGATTGACCGCCTAGAAC
dsPI3Kp85α-T7-R	GGATCCTAATACGACTCACTATAGGGCAAAACCATAGCCATGTT
dsEGFP-F	CGTAAACGGCCACAAGTT
dsEGFP-R	TTCACCTTGATGCCGTTC
dsEGFP-T7-F	GGATCCTAATACGACTCACTATAGGCGTAAACGGCCACAAGTT
dsEGFP-T7-R	GGATCCTAATACGACTCACTATAGGTTCACCTTGATGCCGTTC
**For plasmid construction**	
Src64B-F	CGGGGTACCATGGGCCAGAATATGTGTTGC
Src64B-R	CCGCTCGAGCTACTTGTCATCGTCGTCCTTGTAGTCGAAGGCTGCCGCAGCTGG
PI3Kp85α-F	CGCGGATCCCATGGATTACAAGGACGACGATGACAAGCAGTCAGCAACGGAAAATGTG
pIEx-PI3Kp85α-R	ATAAGAATGCGGCCGCCTAGCTGGCTAGGCGAGCTGGATA
IE1-F	CGGGGTACCATGGCCTTTAATTTTGAAGACTC
IE1-R	CCGCTCGAGCGTACAAAGAATCCAGAAATCTCATC
IE1-Y129F-mut-F	CACGTGGGGGATTTTTTACTTCGCTAG
IE1-Y129F-mut-R	CTAGCGAAGTAAAAAATCCCCCACGTG
